# Broadband Amplification as Tinnitus Treatment

**DOI:** 10.3390/brainsci12060719

**Published:** 2022-05-31

**Authors:** Mie Laerkegaard Joergensen, Petteri Hyvärinen, Sueli Caporali, Torsten Dau

**Affiliations:** 1 Hearing Systems Section, Department of Health Technology, Technical University of Denmark, 2800 Copenhagen, Denmark; petteri.hyvarinen@aalto.fi (P.H.); tdau@dtu.dk (T.D.); 2 WS Audiology, 3540 Lynge, Denmark; sueli.caporali@wsa.com

**Keywords:** tinnitus, hearing aids, amplification, high-frequency hearing loss

## Abstract

This study investigated the effect of broadband amplification (125 Hz to 10 kHz) as tinnitus treatment for participants with high-frequency hearing loss and compared these effects with an active placebo condition using band-limited amplification (125 Hz to 3–4 kHz). A double-blinded crossover study. Twenty-three participants with high-frequency (≥3 kHz) hearing loss and chronic tinnitus were included in the study, and 17 completed the full treatment protocol. Two different hearing aid treatments were provided for 3 months each: Broadband amplification provided gain in the frequency range from 125 Hz to 10 kHz and band-limited amplification only provided gain in the low-frequency range (≤3–4 kHz). The effect of the two treatments on tinnitus distress was evaluated with the Tinnitus Handicap Inventory (THI) and the Tinnitus Functional Index (TFI) questionnaires. The effect of the treatment on tinnitus loudness was evaluated with a visual analog scale (VAS) for loudness and a psychoacoustic loudness measure. Furthermore, the tinnitus annoyance was evaluated with a VAS for annoyance. The tinnitus pitch was evaluated based on the tinnitus likeness spectrum. A statistically significant difference was found between the two treatment groups (broadband vs. band-limited amplification) for the treatment-related change in THI and TFI with respect to the baseline. Furthermore, a statistically significant difference was found between the two treatment conditions for the annoyance measure. Regarding the loudness measure, no statistically significant differences were found between the treatments, although there was a trend towards a lower VAS-based loudness measure resulting from the broadband amplification. No changes were observed in the tinnitus pitch between the different conditions. Overall, the results from the present study suggest that tinnitus patients with high-frequency hearing loss can experience a decrease in tinnitus-related distress and annoyance from high-frequency amplification.

## 1. Introduction

Tinnitus is the perception of phantom sounds that do not originate from external sources [1]. Tinnitus can be described as either being ‘objective’ or ‘subjective’. Objective tinnitus originates from a known source, such as blood vessels or muscle contractions, and can typically be treated surgically [1]. It has been estimated that objective tinnitus represents only approximately 5% of all tinnitus cases, whereas subjective tinnitus, where the source of the tinnitus perception is unknown [1], is much more common (95%). The present study is focused on subjective tinnitus. There have been large differences in the reported prevalence of tinnitus in the general population, ranging from 5.1% to 42.7% of the population [2]. For a subgroup of these people, the perception of their tinnitus is troublesome, persistent and has a severe impact on their quality of life due to sleep deprivation, concentration difficulties, stress, anxiety and depression [3]. Unfortunately, no routine treatment is currently available for tinnitus sufferers, due to difficulties with the assessment of tinnitus, placebo effects, poorly designed studies and the heterogeneity of tinnitus [4]. Tinnitus heterogeneity reflects the large variability of factors associated with tinnitus, including differences in risk factors (e.g., ear pathologies, hearing loss, aging), perception (e.g., loudness, pitch, localization), severity (e.g., distress, annoyance) and comorbidities (e.g., hyperacusis, anxiety) within the tinnitus population [5].

Among the most common comorbidities of tinnitus is hearing loss. However, the association is not straightforward since not all people with hearing loss experience tinnitus, and some people with tinnitus have clinically normal hearing based on a standard audiogram [4]. Several studies have investigated the relationship between hearing loss and tinnitus. Some studies suggest a relationship between the tinnitus pitch and the frequency region with hearing loss [6,7,8,9,10,11] while others suggest a relationship between the tinnitus pitch and the audiogram edge frequency, where the edge frequency is typically defined as the frequency where the hearing loss abruptly increases [12,13]. Finally, some studies fail to report a relationship between the tinnitus pitch and either the edge frequency or the maximum hearing loss frequency [14]. It has been argued that for hearing-impaired tinnitus participants, the presence of hearing loss might contribute to the perceived distress [15].

Further support for the association between hearing loss and tinnitus has been found in studies investigating sound therapy as a possible treatment. Schaette et al. [16] investigated whether acoustic stimulation treatments were more effective when the tinnitus pitch was within the stimulated frequency range than when the tinnitus pitch was outside the stimulated frequency range. They examined the effect of the amplification on tinnitus using self-reported visual analog scales (VAS) and found a significant reduction in tinnitus loudness and severity in the group of participants whose dominant pitch was within the frequency range of the amplification. Similarly, McNeill et al. [17] found a larger effect of the use of HAs for participants that had good low-frequency hearing and where the tinnitus pitch fell into the range of the HAs. In contrast, Moffat et al. [18] did not find any benefit from extended high-frequency amplification for a group of tinnitus patients with a dominant tinnitus pitch at, or above, 4 kHz. The extended high-frequency condition amplified sounds from 250 Hz to 8 kHz, whereas the standard amplification provided gain in the range from 250 Hz to 6 kHz. However, the three studies considered different outcome measures (VAS vs. tinnitus reaction questionnaire vs. tinnitus psychoacoustics), making a direct comparison of the results difficult. Finally, several studies investigated whether tinnitus could be temporarily induced in healthy normal hearing participants [19,20]. The participants were asked to wear earplugs continuously in one ear for several days. The earplugs provided an attenuation similar to a mild high-frequency hearing loss. The majority of participants reported phantom sounds while using the earplugs and, in all cases, the phantom sounds disappeared relatively shortly after the earplugs were removed. This suggested that removing parts of the sensory input can lead to the perception of tinnitus.

Together, these findings suggest that the restoration of peripheral auditory input might reduce tinnitus distress. Two mechanisms have been suggested to cause the benefits of hearing aid (HA) amplification: First, it makes patients pay less attention to their tinnitus, and second, it amplifies sounds and voices, such that the interfering effect of tinnitus on other sounds is less dominant [21]. Thus, the HAs might help the habituation process, where one’s emotional reaction to and awareness of tinnitus is gradually reduced by reducing the attention paid to tinnitus. Furthermore, since tinnitus very often co-exists with hearing loss, the sensory deprivation caused by hearing loss can lead to neural changes and/or neuronal hyperactivity in the auditory pathway [21] whereas these changes can be reversed with sound stimulation. It has therefore been proposed that HAs may help restore normal or close-to-normal neural activity in the auditory pathway by inducing plasticity [21].

Even though the effect of HA amplification on tinnitus has been studied intensely over the last 70 years, recent systematic reviews identified only seven randomized controlled trials (RCTs) that investigated the effect of sound therapy [22,23]. In these reviews, sound therapy was provided by three different types of devices: (1) Amplification-only devices, (2) sound generators (SGs) that do not provide amplification but, instead, play a sound that is more pleasant and acceptable than tinnitus to either mask the tinnitus or shift attention away from it and (3) combination devices that provide both amplification and generate additional sound. The review studies concluded that not enough evidence was available to support or reject the hypothesis that HAs are beneficial as a standard treatment for tinnitus, nor is there sufficient evidence to recommend one device (HAs, SG or combination HAs) over another [22,23]. Four of the seven studies compared HAs with combination devices and similar positive effects were found for the two treatments based on questionnaire scores [24,25,26,27]. One study compared two different treatments that included both behavioral therapy and the use of a sound therapy device. In the first treatment, a behavioral therapy was provided together with HAs, while in the other treatment, a behavioral therapy was provided with SG. No significant differences were found in terms of VAS scores or Tinnitus Handicap Inventory (THI) scores when comparing the treatments [28]. Furthermore, Erlandsson et al. [29] compared SG devices to placebo devices and did not find a difference between the groups in terms of tinnitus intensity based on a 10-point scale. Finally, two studies compared the effect of amplification only to control groups where participants were either on a waiting list or practiced relaxation at home. Whereas Melin et al. [30] only reported a significant improvement in hearing function but not in terms of tinnitus VAS scores, Zhang et al. [31] found improvements in both hearing function and self-reported tinnitus symptom severity. However, even though the studies indicated positive trends of HAs as tinnitus treatment, it was concluded that the quality of the studies was limited due to the lack of blinding and small sample sizes. It was argued that future studies should make use of the international standards for tinnitus trials [32] and should include both self-reported benefits as well as psychoacoustic measures [22,23].

The present study investigated the effect of the frequency range of HA amplification on tinnitus distress, loudness and annoyance using a cross-over design. It was evaluated whether the effect of broadband (125 Hz to 10 kHz) amplification would provide a larger decrease in tinnitus loudness, tinnitus-related stress and annoyance compared to band-limited amplification (125 Hz to 3–4 kHz). It was hypothesized that broadband amplification would cause a larger improvement in terms of tinnitus distress, loudness and annoyance compared to the band-limited amplification because only in the broadband condition did the HAs provide compensation for hearing loss. Furthermore, it was tested whether broadband amplification changed the percept of the tinnitus more than the band-limited amplification. To measure the effectiveness of the two treatments, both psychoacoustic measures and self-reported measures were included.

## 2. Materials and Methods

### 2.1. Participants and Sample Size

**Figure 1:**
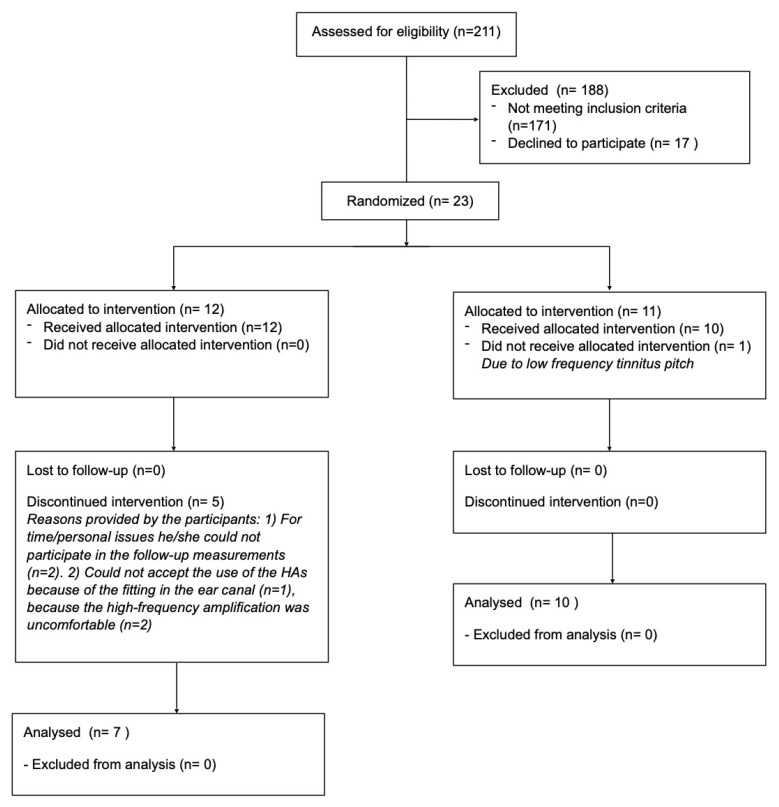
Overview of the participant selection.

Twenty-three participants were included in the study, and 17 participants (mean age of 55.4 ± 12.3 years, range between 28 and 70 years, 6 females, 11 males) with chronic tinnitus finished the study. The 6 dropout participants left the study before the 2nd measurement and their baseline data were not included in the results of the study. An overview of the participant selection process can be found in Figure 1.

The tinnitus duration spanned from 1 year and 10 months to “as long as I remember” (the age of the participant was used for the calculation) with a mean estimated duration of 16.0 ± 13.6 years. The participants located their tinnitus in either the right ear, the left ear, in both ears equally or inside the head. The distribution of the main tinnitus location was as follows: Right ear (11.8%), left ear (23.5%), both ears equally (47.0%) and in the head (17.7%). The participants selected between 1 and 6 words to describe their tinnitus perception, and the distribution was as follows: Hissing (*n* = 9), high voltage wire (*n* = 8), steam whistle (*n* = 7), sizzling (*n* = 6), ringing (*n* = 5), cricket-like (*n* = 3), whistle (*n* = 2), ocean roar (*n* = 2), other (Pure tone from TV pause screen, squeal; *n* = 2), pulsating (*n* = 1), bells (*n* = 1), ticking (*n* = 1) and buzzing (*n* = 1).

**Figure 2:**
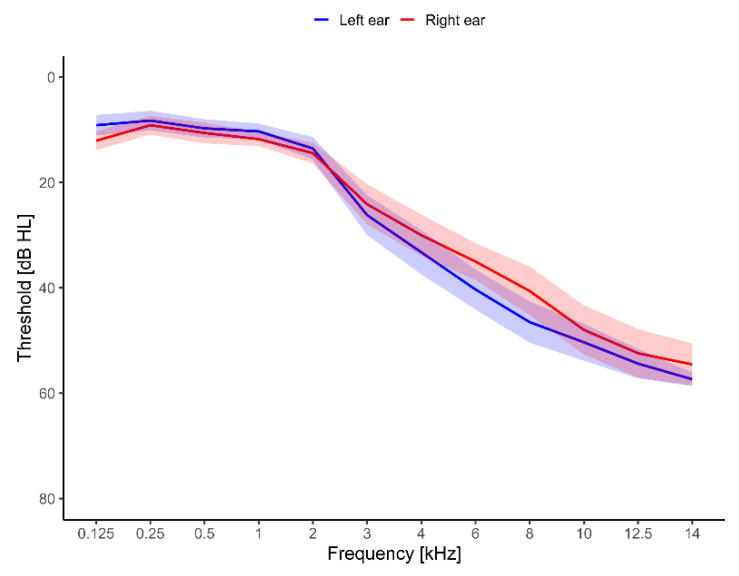
Hearing thresholds were measured from 125 Hz to 14 kHz. Results were averaged over each ear and shown with mean ± SEM. The red line is the average threshold for the right ear, while the blue line is the average threshold for the left ear.

All participants had normal hearing thresholds (≤25 dB HL) up to 2000 Hz and hearing loss at frequencies above 2 kHz. Figure 2 shows the hearing thresholds averaged across the 17 participants. The average threshold from 125 Hz to 2 kHz was between 8 and 15 dB HL. The average threshold for the frequencies from 3 kHz to 14 kHz spanned from 24.1 dB HL to 57.4 dB HL. The average hearing thresholds were symmetrical, with the largest difference of 5.9 dB between the left and the right ear at 8 kHz. The participants’ loudness sensitivity was evaluated with the hyperacusis questionnaire [33]. Their average baseline score was 14.9 ± 1.6 points.

This longitudinal study required the use of HAs for 24 weeks and implied 3–5 visits to the university. Participants who had objective or pulsatile tinnitus, or suffered from Ménière’s disease, otosclerosis, stapedectomy, stapedotomy or tympanoplasty, were excluded from the study. Only inexperienced HA users were considered, i.e., all participants who had used HAs within the past 12 months were excluded from the study. No participants were excluded due to “adverse events”, such as increases in tinnitus distress due to the study treatment, worsening of pre-existing conditions or symptoms in the early phase of the study. However, two participants finished the band-limited treatment preliminary (after approx. 8 weeks) due to increases in tinnitus distress and loudness.

The participants were recruited between December 2018 and July 2020 from multiple sites: The local volunteer database at the Hearing Systems Section at the Technical University of Denmark (DTU), dedicated trial websites and leaflets placed at Centers for Communication Disorders and hearing clinics in the Greater Copenhagen area. The study took place at the Hearing Systems Section at the Department of Health Technology, DTU. The study was approved by the Science-Ethics Committee for the Capital Region of Denmark (reference H-16036391) and was conducted in accordance with the Declaration of Helsinki. All participants provided written informed consent before the start of the study. The study was not registered before subject enrollment, as it was not a requirement of the Science-Ethics Committee. The study has since been registered with ClinicalTrials.org (NCT05271825, March 2022). The authors confirm that all related methods and trials for this intervention are registered.

A power calculation was conducted to serve as a starting point for the sample size determination. Schaette et al. [16] investigated the effect of acoustic stimulation and found an effect size of 0.78 for perceived tinnitus-related distress (measured with the Tinnitus Questionnaire) for participants with a tinnitus pitch within the range of the acoustic stimulation. Based on the effect size, the program G*power [34] was used to calculate the sample size of the present experiment. The calculation was based on the difference between two dependent means *t*-tests for two experimental groups. A statistical significance level, alpha, of 0.05 (two-tailed) and a power value (1-beta) of 0.8 yielded a minimum total sample size of 15 participants. In order to account for differences in study designs and outcome measures, the sample size was set more conservatively to 20 participants.

### 2.2. Procedure

The participants first conducted a prescreening, including standard pure-tone audiometry and questions related to the participant’s hearing and tinnitus. The participants fulfilling the inclusion criteria were invited to take part in the main study.

The study had a cross-over design, where participants were asked to wear HAs with two different fitting conditions for 12 weeks each. Participants were randomly assigned to one of two treatment groups: There was a 50% chance of being in a group where the participants wore HAs with broadband amplification (intervention treatment) from week 0 to week 12, after which they switched to HAs with band-limited amplification (control) from week 12 to week 24. In the other group, participants wore HAs with band-limited amplification for the first 12 weeks and afterwards wore HAs with broadband amplification for the remaining 12 weeks.

The study consisted of three main sessions: The first one took place in week 0, the second one in week 12 and the final one in week 24. A test battery, described in detail further below, was performed during each session, and detailed information about these is provided below. The first session lasted approx. 3.5 h and included an introduction with tinnitus information followed by high-frequency audiometry. Thereafter, the participants conducted the tests in the test battery consisting of psychoacoustic measures and questionnaires. Finally, a pair of HAs were fitted to the participants. During the session, the participants were strongly encouraged to take as many breaks as needed. Sessions 2 and 3 lasted approximately 2 h and consisted only of high-frequency audiometry and the test battery.

### 2.3. Hearing Assessment Procedure

Pure-tone audiometry in the frequency range of 125 Hz to 8 kHz was conducted in a sound-proof booth using a standard clinical audiometer (model AS216, Interacoustics A/S, Middlefart, Denmark) and HD200 headphones (Sennheiser GmbH & Co. KG, Wedermark, Germany). High-frequency audiometry in the frequency range from 8 kHz to 14 kHz was conducted with an automated 1-up–2-down staircase procedure using the AFC framework [35] and HDA 200 headphones.

### 2.4. Tinnitus Information

As an introduction to the study, information about the mechanisms of tinnitus, the auditory system, the relation between tinnitus, stress and emotion as well as the habituation process was provided (ca. 30 min). The tinnitus information was based on “Widex Zen Therapy: Managing the Effects of Tinnitus” [36].

### 2.5. Test Battery

#### 2.5.1. Primary Outcomes

Questionnaires

Subjective tinnitus distress was evaluated using two standardized outcome questionnaires, the Tinnitus Handicap Inventory (THI; [37]) and the Tinnitus Functional Index (THI; [38]). The THI contained 25 questions that were answered with ‘yes’ (4 points), ‘sometimes’ (2 points) or ‘no’ (0 points). The THI resulted in a score between 0 and 100 points. Newman et al. [37] suggested that a 20-point difference in the THI score was the minimum change needed for a clinically significant improvement. However, Zeman et al. [39] suggested that a 7-point change in the THI score would be sufficient to denote a reliable clinically significant improvement. The TFI contained 25 questions that were answered on a scale from 0 to 10 or from 0% to 100%. The TFI resulted in a score between 0 and 100 points. A 13-point change in the TFI score has been considered to denote a reliable clinically significant improvement [38].

#### 2.5.2. Secondary Outcomes

Tinnitus spectrum and loudness

The tinnitus spectrum rating method was adapted from Noreña et al. [6]. As the stimuli, either 2-s long pure tones (1st condition) or 2-s long 1/3-octave noise bands (2nd condition) with center frequencies ranging from 125 Hz to 14 kHz were used. The stimuli were generated in MATLAB version 9.6.0.1072779-R2019a (the Mathworks Inc., Natick, MA, USA) with a sampling rate of 48 kHz. The D/A conversion was performed using an RME Fireface UCX sound card (RME GmbH, Chemnitz, Germany) after which the sounds were amplified with a Phonitor Mini headphone amplifier (SPL electronics GmbH, Niederkruechten, Germany). The stimuli were presented monaurally via HDA 200 headphones to the ear corresponding to the loudest tinnitus perception. In case the tinnitus percept was the same in both ears, the stimuli were presented to the ear with the lowest average high-frequency thresholds. All stimuli were presented at the level matched to the tinnitus loudness for a 1 kHz pure tone, but always at or above a 10 dB sensation level (SL).

The stimuli were presented in a randomized order, and each frequency was rated three times. The tinnitus likeness rating was performed in a soundproof booth and the participants were asked to evaluate the question “How much does the stimulus contribute to your tinnitus?” on a Likert-type scale from 0 (corresponding to “not at all like my tinnitus”) to 10 (corresponding to “Identical to my tinnitus”). The participants could also choose a button with “I cannot hear the sound”.

The tinnitus loudness was estimated in two separate trials. First, the participants were asked to match the loudness of their tinnitus to a 1 kHz pure tone by adjusting the level of the tone in 3-dB steps until the perceived levels were equal. The stimuli in the tinnitus likeness rating were presented with the loudness measured in this step. Second, the participants were asked to match the loudness of their tinnitus to the tone at the frequency that provided the highest average score in the tinnitus likeness rating. If two or more frequencies were given the same average likeness rating, the one with the lowest frequency was chosen. The loudness match was repeated three times. The results from the second part of the measurement will be referred to as the tinnitus matching loudness and will be presented below.

Visual analog scales

All participants were asked to rate their tinnitus loudness on a visual analog scale (VAS; [40]) modified for tinnitus patients. The scale spanned from “inaudible” to “very loud”, and the answer was converted to a score from 0–100, with 0 being “inaudible” and 100 being “very loud”. Similarly, the tinnitus annoyance was measured on a VAS scale spanning from “not annoying” to “very annoying”, converted to a score of 0–100.

### 2.6. Hearing-Aid Fitting Procedure

All participants were fitted bilaterally with Widex Evoke Passion 440 Has with instant open ear tips according to the Widex fitting rationale. All fittings were performed using the Widex fitting software Compass GPS (V. 3.4, Widex, Lynge, Denmark). The “wizard” fitting procedure was followed, which consisted of both the feedback test and the Sensogram. The feedback test measures the in-situ available gain before feedback and serves as the initialization point for the feedback cancelling system. The Sensogram is an in-situ audiogram measured with the hearing aids inside the ears. To keep the study double-blinded, all Has were initially fitted with the high-frequency boost feature, which had the extended bandwidth up to 10 kHz turned on. The specific settings of the HA fittings can be found in Table 1.

Follow-up visits for additional tuning were organized according to the individual participant’s needs. Participants were instructed to use the Has for at least 5 to 6 h per day but were encouraged to use them as much as they wished.

Real-ear measurements

All real-ear measurements (REMs) were performed with the Affinity system (V.2.12 Interacoustics A/S, Middelfart, DK). The International Speech Test Signal (ISTS; [42]) with a frequency range from 250 Hz to 10 kHz was used as the stimulus. A probe tube insertion depth of 30 mm was used to improve the reliability of the high-frequency REM [43]. The ‘real ear unaided gain’ (REUG) and the ‘real ear insertion gain’ (REIG) were measured at 55, 65 and 75 dB SPL. The REIG was measured both with the HAs providing gain in the full frequency range (125 Hz to 10 kHz) and with the HAs providing gain in the band-limited region (125 Hz to 3–4 kHz).

**Figure 3:**
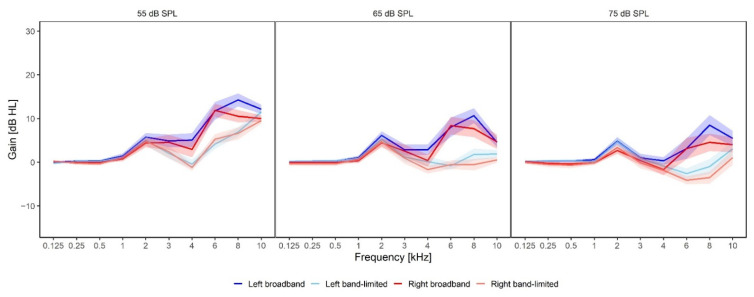
Gain provided by the hearing aids in the two different treatment conditions. The dark blue curve is the gain provided in the left ear in the broadband condition, while the dark red curve is the gain provided in the right ear in the broadband condition. The light blue and red curves are the gain provided in the band-limited condition in the left and right ear, respectively. The gain was measured at 55, 65 and 75 dB SPL with the ISTS.

Figure 3 shows the average gain provided at all frequencies measured with the ISTS at 55, 65 and 75 dB sound pressure levels (SPLs), respectively. On average, no gain was provided at frequencies below 1 kHz, where all participants were considered to have normal hearing (<25 dB HL). The average REIG measured at 55 dB SPL (left panel) was between 4.8 dB and 13.4 dB for the frequencies between 3 and 10 kHz for the broadband condition (dark red/blue functions), whereas it was between −1.2 dB and 11.7 dB for the band-limited condition (light red/blue functions). The largest average gain difference between the broadband and the band-limited amplification was found at 6 kHz for the left ear (blue) where there was an 8.1 dB difference between the conditions. The smallest difference was found at 10 kHz for the left ear (blue) where a difference of 0.4 dB was found. For the REIG measured at 65 dB HL (middle panel), the broadband gain was between 0.7 dB and 10.1 dB, while the band-limited gain was between −2 dB and 2 dB. The largest difference between the conditions was 9.8 dB in the right ear (red) at 6 kHz, while the smallest difference was 1.4 dB in the left ear (blue) at 3 kHz. Finally, the average REIG measured at 75 dB HL (right panel) ranged between 0.1 dB and 8 dB for the broadband condition and between 0.1 dB and 5 dB for the band-limited condition. The largest difference (9 dB) between the two conditions was found at 8 kHz for both the left and right ears. The smallest difference (0 dB) was found at 3 kHz.

### 2.7. Randomization and Blinding

The study had a double-blinded design. The participants were not informed about the settings of their HAs (intervention or placebo). The researcher responsible for the outcome measures did not know which amplification settings of the HAs the participants were using during the 12-week interval. All HAs were initially fitted with broadband frequency amplification. After the fitting and real-ear measurements, a custom-made script was run that randomly allocated participants into groups (intervention or placebo) and selected the proper amplification range. After 12 weeks, a cross-over took place, and participants in the intervention group received the placebo treatment, while participants initially in the placebo group received the intervention treatment. The cross-over took place by running the custom-made script again, with the participant number as an input. All participants received the same information about the expected study outcome. Blocked randomization was used to ensure that the two treatment groups were approximately equal in size.

### 2.8. Statistical Analysis

The analysis included all 17 participants who finished the study. All demographics and baseline characteristics of the included participants were reported with descriptive statistics and included gender, age, tinnitus duration, tinnitus loudness and tinnitus severity.

The experimental and placebo conditions were evaluated with paired *t*-tests. Data are presented as mean ± standard error of the mean (SEM).

## 3. Results

### 3.1. Tinnitus Distress, Loudness and Annoyance Levels in the Baseline Condition

The results obtained regarding tinnitus-related distress, loudness and annoyance in the baseline condition before the treatment with HA amplification are summarized in Table 2 (left column). The tinnitus-related distress was evaluated with both the THI and TFI questionnaires. At baseline, the average THI score was 33.4 ± 5.2 points, which is categorized as a mild tinnitus handicap, while the average TFI score was 38.3 ± 4.9 points. The tinnitus loudness was evaluated with a psychoacoustic loudness matching test and a subjective VAS score. The average baseline tinnitus matching loudness corresponded to a tone played at 13.9 ± 2.4 dB SL, while the average baseline VAS loudness score was 54.5 ± 4.2 points. Finally, the tinnitus annoyance was evaluated with a VAS score and the baseline average was 50.7 ± 5.3 points. There were no statistically significant differences between groups 1 and 2 in the baseline measurements (THI: t(15) = 0.97, *p* < 0.05, TFI: t(15) = 1.73, *p* < 0.05, loudness matching: t(15) = 0.64, *p* < 0.05, VAS loudness: t(15) = 1.90, *p* < 0.05, VAS annoyance: t(15) = 0.67, *p* < 0.05).

### 3.2. Changes in Tinnitus Distress, Loudness and Annoyance after Broadband vs. Band-Limited Amplification

**Figure 4:**
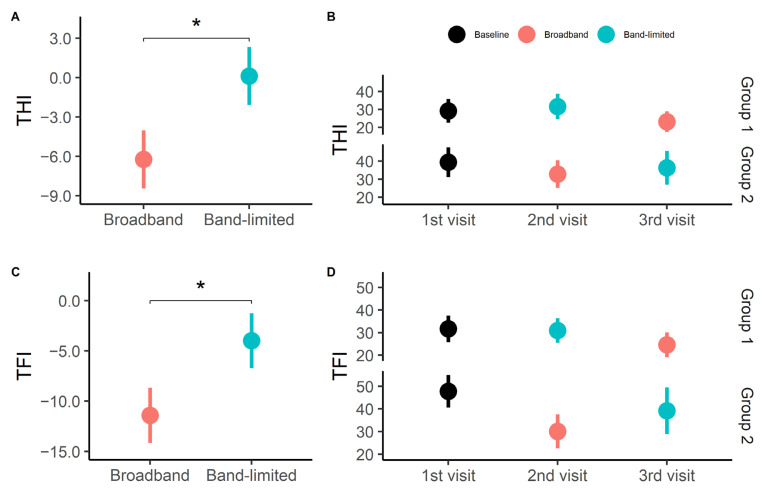
Tinnitus distress. (**A**) Average change in THI scores after broadband and band-limited amplification from baseline. The data from groups 1 and 2 were pooled for both the broadband and band-limited conditions. Averages are presented as mean ± SEM. * *p* < 0.05. (**B**) Average THI scores at each visit subdivided into treatment groups. (**C**) Average change in TFI scores after broadband and band-limited amplification from baseline. The data from group 1 and 2 were pooled for both the broadband and band-limited conditions. Averages are presented as mean ± SEM. * *p* < 0.05. (**D**) Average TFI scores at each visit subdivided into treatment groups.

The primary outcome measure of the study was the tinnitus distress measured using the THI and TFI questionnaires. Figure 4A shows the average change in THI scores from baseline to broadband and band-limited amplification. The average change in THI score after the broadband amplification was −6.2 ± 2.2 points, while the average THI score after the band-limited amplification was 0.1 ± 2.2 points. A statistically significant difference was found between the broadband and the band-limited amplification conditions (t(16) = −2.86, *p* = 0.01124). The broadband amplification resulted in an 18.6% reduction of the THI score relative to the baseline condition and an 18.9% reduction relative to the band-limited condition. Six participants experienced a clinically significant improvement after the use of broadband amplification, while two participants experienced a clinically significant improvement after the use of the band-limited amplification. There was no statistically significant difference between the baseline and band-limited conditions (t(16) = −0.03, *p* < 0.05). Figure 4B shows the average THI scores subdivided into the two treatment groups. When the order of the treatments is considered, the average change in THI score after the broadband amplification was −6.2 ± 5.5 points, while the average THI score after the band-limited amplification was 6.4 ± 5.5 points. A statistically significant difference was found between the two treatment conditions (t(16) = 2.28, *p* = 0.03653).

The corresponding results obtained with the TFI questionnaire are shown in Figure 4C. The average change in TFI score after broadband amplification was −11.4 ± 2.7 points, while the average change in TFI score after band-limited amplification was −4.0 ± 2.7 points. A statistically significant difference was found between the broadband and the band-limited conditions (t(16) = −2.72, *p* = 0.01523). This corresponds, on average, to a 29.8% reduction of the TFI score relative to the baseline condition and a 21.7% reduction relative to the band-limited condition. Five participants experienced a clinically significant improvement after the use of broadband amplification, while one participant experienced a clinically significant improvement after the use of band-limited amplification. There was no statistically significant difference between the baseline and band-limited conditions (t(16) = 1.10, *p* < 0.05). Furthermore, Figure 4D shows the average TFI score for each group, and it can be seen that, regardless of the order of treatments, a reduction was only found after the use of the broadband amplification treatment. When the order of the treatments was considered, the average TFI score after the broadband amplification was −11.4 ± 6.3 points, while the average TFI score after the band-limited amplification treatment was 7.4 ± 6.3 points. A statistically significant difference was found between the two treatment conditions (t(16) = 3.01, *p* = 0.008303).

**Figure 5:**
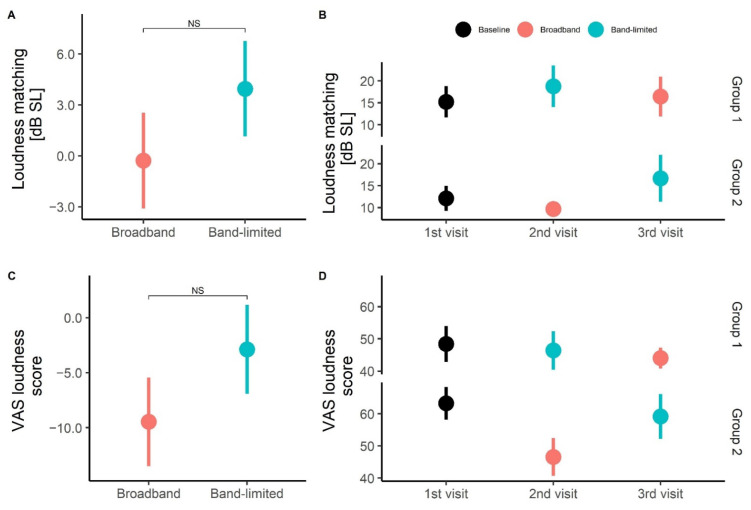
Tinnitus loudness. (**A**) Average change in tinnitus loudness matching after broadband and band-limited amplification from baseline. The data from groups 1 and 2 were pooled for both the broadband and band-limited conditions. Averages are presented as mean ± SEM. (**B**) Average tinnitus loudness matching at each visit subdivided into treatment groups. (**C**) Average change in VAS loudness score after broadband and band-limited amplification from baseline. The data from groups 1 and 2 were pooled for both the broadband and band-limited conditions. (**D**) Average VAS loudness score at each visit subdivided into treatment groups.

The second outcome measure was tinnitus loudness, which was measured both with a psychoacoustic loudness matching test and a subjective VAS score for loudness. The average change in loudness matching for both the broadband and the band-limited conditions is shown in Figure 5A. The average change in level to obtain a loudness match for the broadband condition was −0.3 ± 2.8 dB SL, while the average change in level for the band-limited condition was 4.0 ± 2.8 dB SL. No statistically significant differences were found between the two conditions (t(16) = −1.51, *p* < 0.05). Furthermore, no statistically significant difference was observed between the baseline and band-limited conditions (t(16) = −1.55, *p* < 0.05). Figure 5B shows that the level of the tone matched to the tinnitus loudness remained very similar regardless of the treatment order, and no statistically significant differences were found between the two treatment conditions when the treatment order was taken into consideration (t(16) = 1.13, *p* < 0.05). The average change in level needed to obtain a loudness match for the broadband condition was −0.28 ± 4.0 dB SL, while the average change in level needed for the band-limited condition was 4.2 ± 4.0 dB SL. The average change in VAS loudness score for the broadband condition was −9.5 ± 4.0 points and the average score for the band-limited condition was −2.9 ± 4.0 points (Figure 5C). No statistically significant difference was found between the two conditions, but the results indicated a trend that the broadband amplification resulted in a lower average value than the band-limited amplification (t(16) = −1.51, *p* < 0.05). No statistically significant difference was observed between the baseline and band-limited conditions (t(16) = 0.88, *p* < 0.05). Furthermore, Figure 5D shows the data subdivided into the two treatment order groups. No statistically significant differences were found between the two conditions (t(16) = 1.96, *p* < 0.05). The average change in VAS loudness score for the broadband condition was −9.47 ± 8.2 points and the average score for the band-limited condition was 6.6 ± 8.2 points when the treatment order was considered.

**Figure 6:**
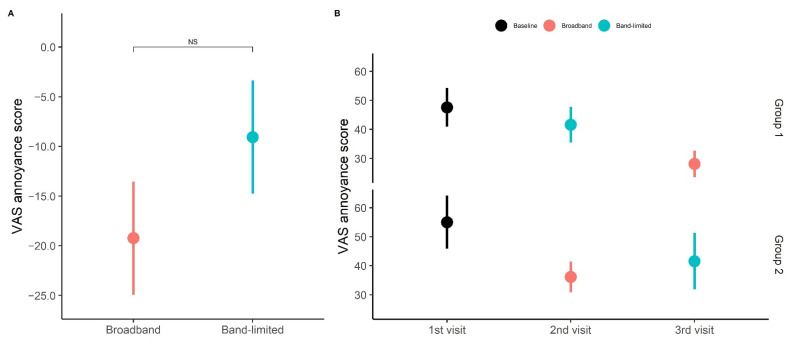
Tinnitus annoyance. (**A**) Average change in VAS annoyance score after broadband and band-limited amplification compared to the baseline. The data from groups 1 and 2 were pooled for both the broadband and band-limited conditions. Averages are presented as mean ± SEM. (**B**) Average VAS annoyance score at each visit subdivided into treatment groups.

In terms of tinnitus annoyance (Figure 6A), the average change in annoyance obtained in the broadband condition was −19.2 ± 5.7 points and −9.1 ± 5.7 points in the band-limited condition. No statistically significant difference was found between the two conditions. However, there was a trend towards a lower average value for the broadband than in the band-limited condition (t(16) = −1.79, *p* < 0.05) and Figure 6B shows that larger reductions were obtained with the broadband amplification than with the band-limited amplification for both treatment groups. Both the broadband and the band-limited conditions were significantly different from the baseline condition (broadband: t(16) = 3.20, *p* = 0.006, band-limited: t(16) = 2.96, *p* = 0.009). A statistically significant difference was found between the two treatment conditions when the order of the treatments was considered (t(16) = 2.60, *p* = 0.01926). The average change in annoyance obtained in the broadband condition was −19.2 ± 11.3 points, while the average change obtained in the band-limited condition was 10.2 ± 11.3 points.

**Figure 7:**
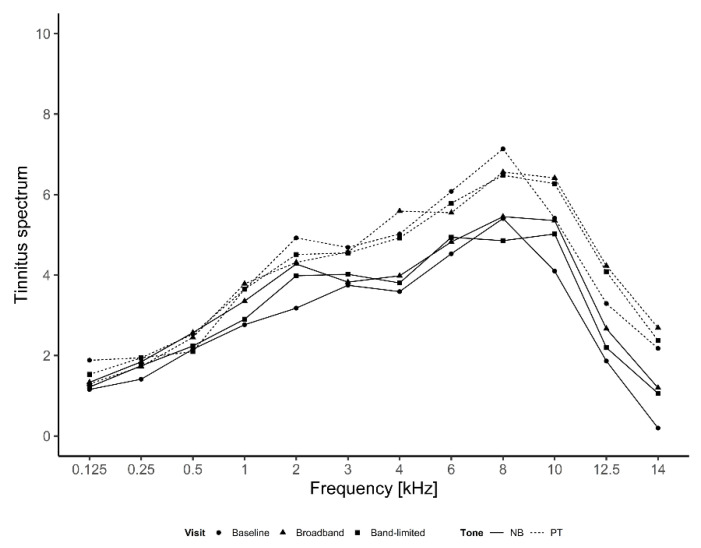
Averaged tinnitus spectrum measured at baseline, after broadband amplification and after band-limited amplification with both pure-tones and narrowband noise.

Finally, the tinnitus spectrum was evaluated in the baseline condition and after treatment with the two amplification conditions. The results are shown in Figure 7. The spectrum scores increased with frequency until 10 kHz, whereafter the scores decreased. This indicates that the frequencies of 4–10 kHz were predominant in the participant’s tinnitus perception. The highest average scores were found at 8 kHz with 7.1, 6.6 and 6.5 for the baseline, broadband and band-limited conditions, respectively. The overall pattern of the spectrum was the same in the different conditions, indicating that the use of broadband and band-limited amplification did not change the pitch perception of the tinnitus. However, the pure-tone sounds were generally scored higher than the narrowband noises for frequencies between 1 and 10 kHz.

**Figure 8:**
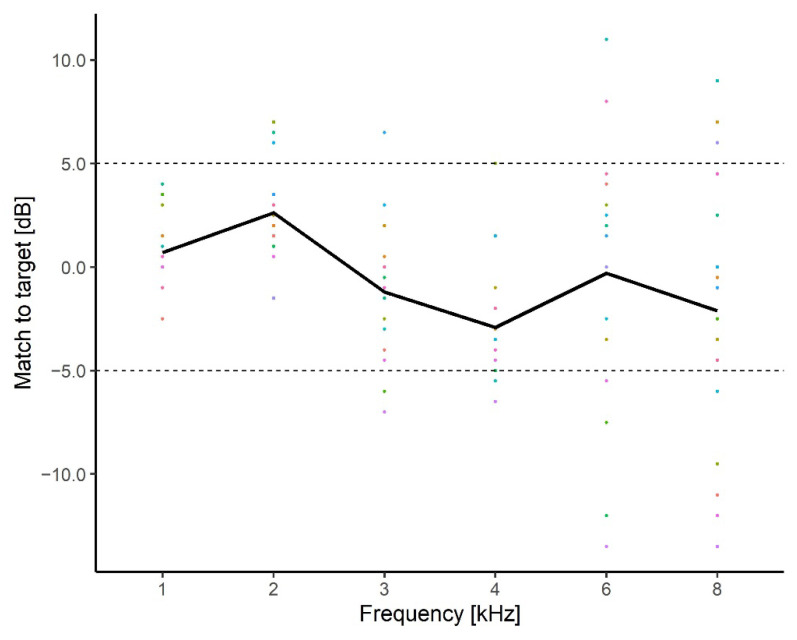
Difference between REIG and the target gains provided by the hearing aid fitting software. The average of the left and right ears is shown. Individual participant data (colored lines) and the average (black line) across participants are shown. The dotted lines represent the +/− 5 dB tolerance recommended at frequencies between 250 Hz and 6 kHz when comparing the REIG to the target value (BSA 2018).

To evaluate whether the improvement with respect to tinnitus distress was related to the amount of gain provided by the HAs, the differences between the REIG measured at 65 dB HL and the target gain provided by the fitting software were calculated. Figure 8 shows the difference between the REIG and the target gain for frequencies between 1 and 8 kHz for each participant (dashed colored lines). The solid black line indicates the average difference between the REIG and target across participants. The average values were within ±5 dB of the target gain as recommended for standard HA fittings. However, the individual REIG deviated up to ±13.5 dB from the target gain at frequencies above 6 kHz.

**Figure 9:**
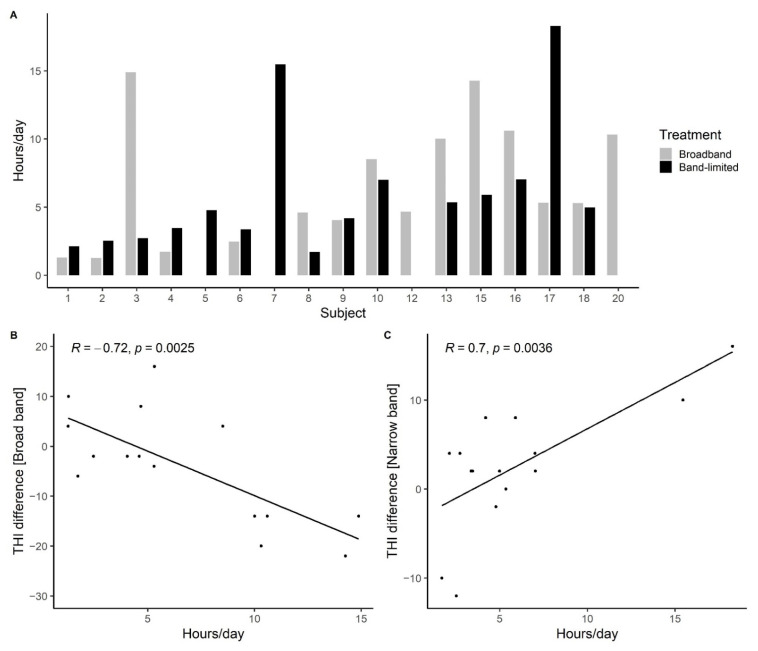
(**A**) Overview of the number of hours/day the hearing aids have been switched on. (**B**) Pearson’s correlation between the THI difference before/after the use of broadband amplification and the amount of hours/day the HAs were active. (**C**) Correlation between the THI difference before/after the use of band-limited amplification and the amount of hours/day the HAs were active.

Figure 9A shows the time the HAs were active. There are four missing data points due to technical issues with obtaining the HA data logs; however, all four participants reported daily use of the HAs. The number of active hours per day ranged from 1.3 h/day for participant 2 in the broadband condition to 18.3 h/day for participant 17 in the band-limited condition. To evaluate whether the active time of the hearing aids was related to the changes in tinnitus distress, the active hours per day were correlated with the THI difference values. Figure 9B shows a moderate negative correlation between the THI difference before and after broadband amplification and the active hours of HA use per day (r(14) = −0.72, *p* = 0.0025). However, a moderate positive correlation was found between the THI difference before and after the use of the band-limited amplification and the active hours of HA use per day (r(14) = 0.7, *p* = 0.0036; Figure 9C). As seen in Figure 9A, for four participants, the HAs were active for more than 12 h per day on average throughout the study period in one of the two treatment conditions. Since the HAs only could register when they were switched on and not when they were being used, it is possible that some participants forgot to switch off the HAs during the night. The correlations were therefore also calculated with the four data points above the 12 h per day removed. After the broadband treatment, a moderate negative correlation was found between the THI improvement and the active hours per day (r(12) = −0.6, *p* = 0.029), while no statistically significant correlation was found between the THI score and the active hours per day after the band-limited treatment (r(12) = 0.46, *p* < 0.05). This indicates that increased use of the HAs throughout the day decreased the THI score, but only when high-frequency gain was provided. When only low-frequency gain was provided, the THI improvement did not depend on the number of hours the HAs were used.

## 4. Discussion

The present study evaluated whether high-frequency amplification has an effect on tinnitus distress, annoyance, loudness and pitch. A statistically significant difference was found between the two treatment groups (broadband and band-limited) for the tinnitus-related distress and annoyance. No statistically significant differences were found between the treatments for the loudness measures; however, a trend that broadband amplification resulted in larger improvements compared to the band-limited condition was observed for the loudness measure obtained with VAS. Furthermore, no statistically significant differences were found in the tinnitus spectrums for the different conditions.

Consistent with previous reviews on the effect of HAs on tinnitus [22,44,45], the present study demonstrated a beneficial effect of wearing HAs on tinnitus distress. However, here, the beneficial effect was only found in connection to the broadband amplification but not in connection to the band-limited amplification. The average decrease in tinnitus distress represented by the THI and TFI questionnaires was not clinically significant following the definitions of Newman et al. [37] and Meikle et al. [38] and was smaller than what was previously reported in Sereda et al. [23]. However, a study from Zeman et al. [39] suggested that smaller THI differences resulted in clinically meaningful changes. Moreover, a reduction of 6 or 7 points in the THI score was identified as the minimal clinically relevant change. Furthermore, no statistically significant differences were found between the psychoacoustic loudness data before and after the HA treatments. However, the obtained average loudness values, corresponding to SLs between 13.7 and 17.9 dB in the different conditions, were consistent with previous findings [46,47]. The loudness was also evaluated on a VAS scale and a 9.4-point difference was found between the baseline and broadband conditions. This decrease was similar to the values reported in previous studies [16,48]. However, Schaette et al. reported an almost twice as large decrease in a subgroup of participants whose tinnitus pitch was within the frequency range of the used HAs. Since this was also the setup used in the present study, similar results were expected.

The smaller improvements observed in the present study compared to previous investigations might, at least partly, be related to the highly controlled study design and the participant inclusion criteria. First of all, since only participants with high-frequency hearing loss and normal hearing at low frequencies were included, the main complaint of the participants was tinnitus and not difficulties with hearing. This might have affected the participant’s willingness to use the HAs for prolonged periods of the day. The data-logging data showed that the majority of participants used the HAs less than 5 h a day. This was 2–4 h less than the averaged data-logging data reported by Henry et al. [25] for new HA users with tinnitus and hearing difficulties. Secondly, the inclusion criteria of only including participants with hearing losses above 3 kHz made it difficult to provide sufficient gain in the high frequencies for seven of the participants due to hardware limitations. Furthermore, the HAs were set with maximum noise reduction and an inexperienced user setting to optimize comfort for the participants. However, these settings might have decreased the effect of the treatment by slightly decreasing the amount of gain provided and by not providing background sounds in quiet situations [41,49]. Finally, all participants who reported suffering from tinnitus and fulfilled the inclusion criteria were included in the study regardless of their baseline tinnitus distress scores. Five of the seventeen participants had a baseline THI score below 18, categorized as “no or slight tinnitus handicap”, which made it difficult to achieve large improvements in distress levels.

Previous reviews described the need for highly controlled procedures to evaluate the effect of HAs on tinnitus perception [22,23]. A major emphasis was therefore put on the study design of the present study to eliminate as many biases as possible. The study was double-blinded to make sure that both the participants and the investigator were not informed about the specific treatment. Furthermore, to avoid confounding covariates and improve the statistical power of the study, a crossover design was chosen. Most importantly, we attempted to include a placebo condition that required the daily use of the HAs but did not provide amplification in the frequency region of the hearing loss. The REM showed a clear difference between the provided gain in the two treatment conditions for frequencies between 3 and 6 kHz, while smaller differences were found at and above 8 kHz. Furthermore, none of the participants reported that they experienced non-active HAs after the band-limited treatment had been finalized, regardless of the order of treatment. This indicates that the band-limited condition can be seen as a valid placebo condition that provides the sensation of treatment, but without the benefits of the high-frequency amplification.

The participant population was deliberately chosen to be very homogenous with regards to the hearing thresholds to ensure the active placebo condition. However, the general tinnitus population is very heterogeneous [1] and it is, therefore, difficult to generalize the findings of this study to patients with, e.g., low-frequency hearing losses or more severe types of hearing loss. Furthermore, due to the high-frequency hearing losses of the included participants, it was difficult to provide the desired amount of high-frequency amplification. For nearly half of the participants, it was not possible to provide as much gain as suggested by the HA fitting software due to hardware limitations. In addition, several participants decided to leave the study prematurely. It is possible that the participants who withdrew from the study were more likely to be dissatisfied with the intervention [50]. Finally, the gain provided in the two experimental conditions was evaluated with REM, and due to the study design, the high-frequency gain was especially important. The test–retest reliability of REM has previously been validated up to 6 kHz [51]. However, at higher frequencies, the probe tube placement requires very close proximity to the tympanic membrane to avoid interactions with standing waves [52]. It is therefore possible that the REIG measured at 8 and 10 kHz has larger deviations from the actual gain provided by the HAs than the REIG measured at and below 6 kHz.

Although the current study suggests that providing gain in frequency regions with hearing loss is a beneficial tinnitus treatment, it also shows that the treatment is not sufficient to fully remove the tinnitus percept. Furthermore, large individual differences in the effects of the treatment have been observed. This indicates that the HA treatment can be further improved and individualized. As mentioned above, for some participants, there were limitations in terms of the amount of gain that could be provided by the HAs at high frequencies. It would therefore be interesting to further develop the possibilities of providing high-frequency gain. Furthermore, in recent years, there has been an increasing interest in personalizing HA fittings based on ‘auditory profiles’ that are based on information beyond the standard audiogram to characterize the perceptual consequences of an individual person’s hearing loss, such as loudness and speech-in-noise perception [53,54,55]. It would be interesting to investigate whether these types of personalized HA fittings would be beneficial for the tinnitus population.

## 5. Conclusions

In summary, the present study found a statistically significant decrease in tinnitus distress and annoyance after a broadband amplification treatment compared to the baseline condition. A similar trend was found for the tinnitus loudness measured with VAS. Furthermore, increased usage of the HAs suggested larger improvements in tinnitus distress for the broadband amplification treatment than for the band-limited amplification treatment. The results indicate that HA amplification can help reduce tinnitus distress. However, individual differences were found in all conditions, suggesting that further optimization and personalization of HA fittings might be needed. Furthermore, the results were obtained in a relatively small sample size of 17 participants with mild-to-moderate hearing loss. The tinnitus population is very heterogeneous both regarding hearing loss and tinnitus perception. More knowledge is therefore required regarding the effect of HA treatment for tinnitus patients with more severe hearing loss as well as those with low-frequency hearing loss.

## Figures and Tables

**Table 1:** Overview of the Widex Evoke Passion 440 hearing aid settings used in the present study.

Function	Setting
Fitting rationale	Widex, new user open fit *
Broadband frequency range	125 Hz to 10 kHz **
Narrowband frequency range	125 Hz to 3–4 kHz ***
User mode	Balanced
Soft-level noise reduction	Max reduction (reduce the soft-level noise with up to 11 dB)
Microphone mode	Hd Locator with Digital Pinna
Speech and noise mode	Real-time speech Enhancer
Wind noise attenuation	Enabled
Impulse sound mode	TruSound Softner enabled
Feedback-cancelling mode	SuperGain (prioritizes sound quality by reducing feedback with up to 9 dB and increase amplification with up to 3 dB)

* The new user open fit Widex rationale is approximately 2 dB lower than the experienced open fit rationale. For more information please see [41]. ** The broadband frequency range was selected due to hardware limitations. *** The band-limited frequency range depended on the subjects’ hearing loss. Subjects with normal hearing up to 2 kHz had a band-limited frequency range from 125 Hz to 3 kHz, while subjects with normal hearing up to 3 kHz had a band-limited frequency range from 125 Hz to 4 kHz.

**Table 2:** Overview of tinnitus severity at baseline and after treatment with broadband and band-limited amplification. The data from groups 1 and 2 were pooled for both the broadband and band-limited conditions.

	Baseline (Mean ± SEM)	Broadband (Mean ± SEM)	Band-Limited (Mean ± SEM)
THI	33.4 ± 5.2 points	27.2 ± 4.6 points	33.5 ± 5.5 points
TFI	38.3 ± 4.9 points	26.9 ± 4.3 points	34.3 ± 5.2 points
Loudness matching	13.9 ± 2.4 dB SL	13.7 ± 2.8 dB SL	17.9 ± 3.4 dB SL
VAS loudness	54.5 ± 4.2 points	45.1 ± 3.0 points	51.7 ± 4.7 points
VAS annoyance	50.65 ± 5.32 points	31.4 ± 3.5 points	41.6 ± 5.2 points

## Data Availability

The data presented in this study are available on request from the corresponding author.
